# Journal Citation Report 2024 and Impact Factor of Pakistani Biomedical Journals: Grading impact and analysis

**DOI:** 10.12669/pjms.40.7.10399

**Published:** 2024-08

**Authors:** Sultan Ayoub Meo, Shaukat Ali Jawaid

**Affiliations:** 1 Sultan Ayoub Meo, MBBS, Ph.D, FRCP Professor and Consultant, Department of Physiology, College of Medicine, King Saud University, Riyadh, Saudi Arabia. E-mail: smeo@ksu.edu.sa sultanmeo@hotmail.com; 2 Shaukat Ali Jawaid Chief Editor, Pakistan Journal of Medical Sciences, Karachi, Pakistan. E-mail: pulse@pulsepakistan.com pjms@pjms.com.pk

The global scholarly journals ranking list, Journal Citation Report (JCR), was released by Clarivate, a leading global institute, on June 20, 2024. This report provides comprehensive information about all the global journals indexed in Science Citation Indexed Expanded (SCIE), the Web of Science Core Collection; and the Emerging Sources Citation Index (ESCI). The academic journals are ranked to enable academic institutions, researchers, publishers, and policymakers to assess and understand the significance and scientific worth of journals in the global research landscape. There are 21,848 journals including 5,800 open-access journals. These academic journals are from 113 countries, across 254 categories. This includes 14,090 science journals, 7,321 social science journals and 3,304 arts & humanities journals, and 544 journals receive a Journal Impact Factor for the first time.^1^

Once again, the leading science journals including CA-A CANCER JOURNAL FOR CLINICIANS (503.1), Nature Reviews Drug Discovery (122.7), Lancet (98.4), New England Journal of Medicine (96.2), British Medical Journal (93.6). Nature (50.5), and Science (44.7) achieved a high place in the Impact Factor race worldwide.^2^ These are the journals which are leading the scholarly world, and publishing articles in these journals is the dream of any academician and researcher.

While analyzing the academic journals from Pakistan listed by Clarivate 2024, it is unfortunate to note that out of 264 universities, and 380 academic journals, only 32 (8.42%) journals from various fields of science and social sciences achieved a place in Clarivate. Among these 32 journals, 11 journals are indexed in Science Citation Indexed Expanded (SCIE), and 21 journals are indexed in Emerging Sources Citation Index (ESCI).3 These 21 ESCI journals are still struggling to shift from ESCI to SCIE, whereas some still cannot place a position in the SCIE in the last five years. Out of these 32 Clarivate indexed journals, 20 journals (10 in SCIE and 10 in ESCI) are from biomedical sciences (Table-I).

**Table-I:** Biomedical Academic Journals from Pakistan indexed in Clarivate with Impact Factor and Quartile Ranking.

Name of the Journal	Impact Factor	Quartile Ranking	JIF Percentile
**Journals Indexed in Science Citation Indexed Expanded (SCIE)**			
Pakistan Veterinary Journal	3.8	Q1	94.9
**Pakistan Journal of Medical Sciences**	**1.2**	**Q2**	**54.0**
Journal of the Pakistan Medical Association	0.8	Q3	41.7
JCPSP-Journal of the College of Physicians and Surgeons Pakistan	0.7	Q3	36.5
Pakistan Journal of Agricultural Sciences	0.7	Q3	35.4
Journal of Animal and Plant Sciences	0.6	Q3	29.8
Pakistan Journal of Botany	0.9	Q4	22.1
Pakistan Journal of Pharmaceutical Sciences	0.7	Q4	10.6
Pakistan Journal of Zoology	0.5	Q4	9.7
International Journal of Pharmacology	0.3	Q4	3.0
**Journals indexed in Emerging Sources Citation Index (ESCI**) **Note: These journals are still under evaluation and have not shifted to SCIE (June 2024)**			
Asian Journal of Agriculture and Biology	1.6	Q2	62.4
Gomal Journal of Medical Sciences	0.5	Q3	30.0
Rawal Medical Journal	0.4	Q3	28.2
Advancements in Life Sciences	0.9	Q3	26.1
Khyber Medical University Journal-KMUJ	0.2	Q4	17.7
Anesthesia Pain and Intensive Care Journal	0.2	Q4	14.8
Pakistan Heart Journal	0.2	Q4	7.0
Annals of King Edward Medical University Lahore Pakistan	0.1	Q4	7.8
Journal of Pioneering Medical Sciences	0.1	Q4	7.8
Journal of the Liaquat University of Medical and Health Sciences	0.1	Q4	0.9

***Note:*** Quartile Ranking, JIF Percentile and Impact Factor of Pakistani Biomedical Journals.^3^

**Fig.1:**
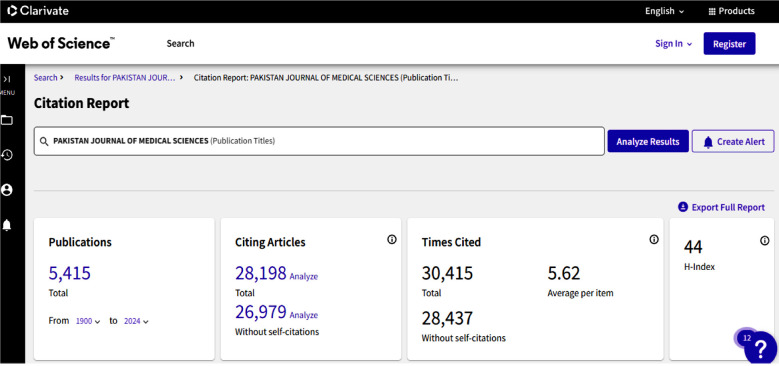
Citation Report of Pakistan Journal of Medical Sciences.^3^

**Fig.2:**
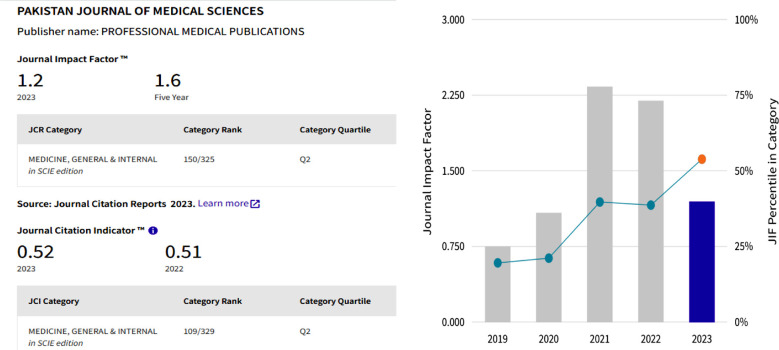
Impact Factor and Quartile Ranking of Pakistan Journal of Medical Sciences.^3^

**Fig.3:**
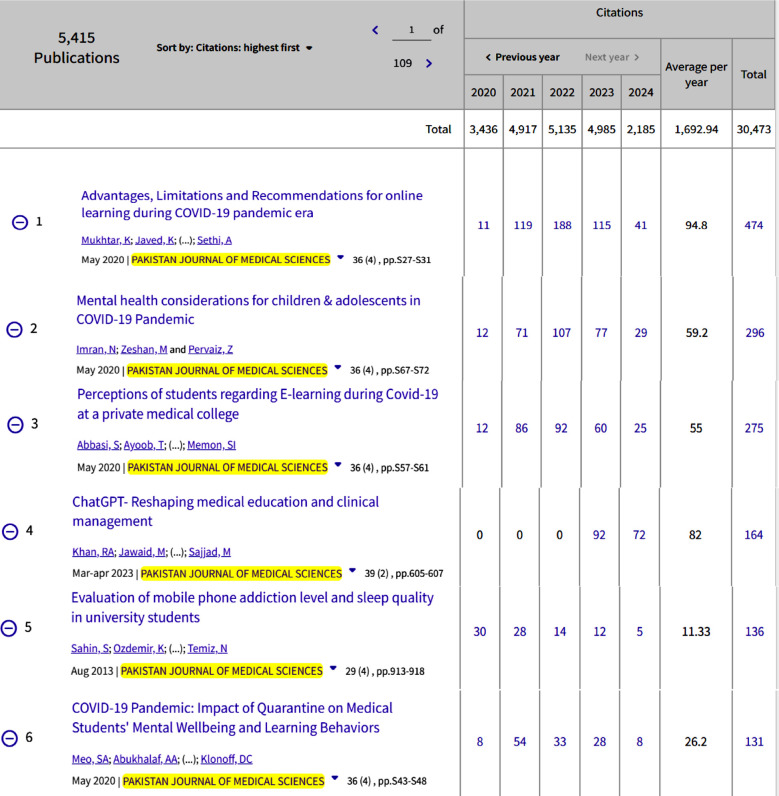
Most cited articles published in the Pakistan Journal of Medical Sciences.^3^

The more painful situation is the Impact Factor (IF) and Quartile ranking of these Pakistani journals in Global Science. Only two biomedical journals achieved a place in quartile ranking 1 and 2, Pakistan Veterinary Journal IF 3.8 with a quartile ranking 1 (Q1); and Pakistan Journal of Medical Sciences IF 1.2 with a quartile ranking of 2 (Q2). However, the remaining 08 journals’ IF was less than 1 (04 journals under Q3 and 04 journals achieved a place in Q4). However, 10 Pakistani biomedical journals are listed in the ESCI, and these journals’ impact factor is rotating 0.1-1.6, only one journal is listed in Q2 with IF 1.6, three journals in Q3, and six journals ranked under Q4 (Table-I). The citation Report of the Pakistan Journal of Medical Sciences is reflected in Fig.1-2, while some of the recent most cited articles are highlighted in Fig.3.

In medical and health sciences, only one journal, the Pakistan Journal of Medical Sciences achieved an IF 1.2 with quartile ranking two (Q2). It established a platform to publish quality research but it is yet a dire need for the science community to publish quality work to fascinate the international science community and enhance the research visibility in the global science to upsurge the IF and quartile ranking One (Q1). The cornerstone of any nation’s progress and prosperity is based on the twin pillars of education and research which establishes the foundation of a progressive future. The nations which invest in education and research and develop a culture of innovation tend to experience accelerated growth, and improved quality of life. However, nations which neglect these areas often find themselves mired in poverty, social strife, and stagnation. The path to Pakistan in its national interest is paved with the bricks of education and research and development on merit-based culture in the universities and research institutes these are essential for the progress and prosperity of nations.

While we in Pakistan Journal of Medical Sciences are delighted at the fact that it has earned a place in Q2 category which is an important achievement, but the Impact Factor decreased. It is also a fact that the Impact Factor of most of the global journals has been reduced because of the new policy by Clarivate. For example, it reflects the unified ranking of two hundred twenty-nine science and social science categories. However, as a policy, we have decided that in future to maintain the standard of the journal and ensure that high-quality papers are accepted for publication, only studies with some innovation and those which have increased chances of further citation will be accepted for further processing. However, papers which have already been accepted for further processing will not be affected with this decision.

We are mindful of the difficulties faced by authors who are keen to publish their research in Pakistan Journal of Medical Sciences, but we have our own limitations as regards human resource and financial constraints. We practice Open Peer Review system and peer review takes lot of time. First of all, it is not an easy task to find excellent quality reviewers and secondly, they have to be sent reminders repeatedly since they too are very busy academicians. We are trying to clear the backlog first and then will accept further manuscripts in the light of the new revised policy. We hope that the authors will realize our difficulties and problems too.

A strange situation has now emerged. While we have too many submissions and we cannot entertain all those though some of them include excellent quality manuscripts as we do not wish to keep the authors waiting for too long. Timely publication of research is essential otherwise it will lose its impact. On the other hand, Pakistan Medical & Dental Council has approved quite a few medical journals after detailed assessment and review by the Journals Assessment and Evaluation Committee and they are short of papers. Authors particularly from Pakistan are advised to submit their manuscripts to these journals, details can be found on the PM&DC website which will ensure early publication of their manuscripts and it will also help the journals in timely publication.

We receive manuscripts from across the world, but the number of submissions from some Asian countries is enormous. We are careful to include only quality research. We will encourage authors from as many countries as possible. However, we also have to be careful of self-citations as well. Some authors do cite papers published in Pakistan Journal of Medical Sciences presuming it might help in acceptance of their papers but to ensure transparency, we request the authors that only relevant papers should be cited.

Yet another issue that we have noted is some authors get confused and are easily trapped by journals having similar names to Pakistan Journal of Medical Sciences. When they find out, it is too late. We expect authors to be careful while making submissions and check the website carefully while the regulatory authorities like Higher Education Commission and Pakistan Medical & Dental Council should refuse to recognize these journals with similar names. It will save many authors from being trapped as it also brings a bad name for the country. It is also in the interest of these journals to have their own identity.
